# Recent Advances in Optical Fiber Enabled Radiation Sensors

**DOI:** 10.3390/s22031126

**Published:** 2022-02-01

**Authors:** Jing Zhang, Yudiao Xiang, Chen Wang, Yunkang Chen, Swee Chuan Tjin, Lei Wei

**Affiliations:** 1 School of Mechanical Engineering and Electronic Information, China University of Geosciences, 388 Lumo Road, Wuhan 430074, China; 1202120782@cug.edu.cn (Y.X.); 1202120758@cug.edu.cn (C.W.); cyk@cug.edu.cn (Y.C.); 2 School of Electrical and Electronic Engineering, Nanyang Technological University, 50 Nanyang Avenue, Singapore 639798, Singapore; esctjin@ntu.edu.sg

**Keywords:** optical fiber radiation sensor, multi-material fiber, single crystal optical fiber, radiation-induced attenuation, radiation-induced luminescence, radiation-induced grating wavelength shift

## Abstract

Optical fibers are being widely utilized as radiation sensors and dosimeters. Benefiting from the rapidly growing optical fiber manufacturing and material engineering, advanced optical fibers have evolved significantly by using functional structures and materials, promoting their detection accuracy and usage scenarios as radiation sensors. This paper summarizes the current development of optical fiber-based radiation sensors. The sensing principles of both extrinsic and intrinsic optical fiber radiation sensors, including radiation-induced attenuation (RIA), radiation-induced luminescence (RIL), and fiber grating wavelength shifting (RI-GWS), were analyzed. The relevant advanced fiber materials and structures, including silica glass, doped silica glasses, polymers, fluorescent and scintillator materials, were also categorized and summarized based on their characteristics. The fabrication methods of intrinsic all-fiber radiation sensors were introduced, as well. Moreover, the applicable scenarios from medical dosimetry to industrial environmental monitoring were discussed. In the end, both challenges and perspectives of fiber-based radiation sensors and fiber-shaped radiation dosimeters were presented.

## 1. Introduction

**Figure 1:**
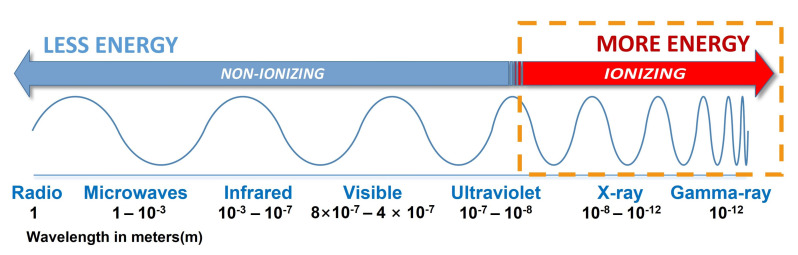
Ionizing and non-ionizing electromagnetic radiation.

Ionizing radiation science has many applications, ranging from high energy physics, industrial, and medical treatment, to space exploration and national defense. Ionizing radiation consists of electromagnetic waves with sufficiently high energy to ionize atoms and molecules, including the higher energy ultraviolet, the X-rays, and gamma rays (Figure 1) [1,2]. Because of the high energy effects of ionizing radiation on environment and human body, accurate radiation monitoring and dosimetry devices are indispensable. Therefore, semiconductor sensors, photoelectric sensors, and other integrated sensors based on various principles have been widely studied for radiation sensing applications. However, these sensors are easily interfered by electromagnetic fields or other radiation sources, making them malfunction in many scenarios.

Nowadays, optical fiber-based radiation sensors show several unique advantages owing to their material and structural features when being used in radiation sensing [3,4,5,6,7,8]. Firstly, optical fiber sensors are small in size, light in weight, long in the sensing distance, and flexible to bend. The diameter of commonly used optical fiber is 125 μm and the diameters of advanced multi-material multi-functional fibers are a few hundred microns, making the whole fiber-based sensors occupy diameters less than 1 mm. In addition, the fibers are flexible and bendable, making them ideal for attaching to curved surfaces. Therefore, optical fiber sensors can be inserted into narrow, curved, and complex environments, such as the inside of aircraft or the human body, which are suitable for radiation sensing and monitoring. Moreover, fiber-derived sensors provide a much wider monitoring range compared to conventional integrated sensors. It is difficult for the conventional integrated sensors to monitor a large area with the point-by-point detection, but for optical fiber sensors being distributed over a very long distance over kilometers, sensing at every point along the fiber becomes possible with the optical time-domain reflectometer (OTDR) technology [6,9]. Secondly, with the prosperous development of the fiber fabrication technology and related material engineering, the optical fiber has been developed from the core-cladding structure based on quartz glass to composite structures with multi-materials, including semiconductors, metals, doped silica glasses, oxide glasses, chalcogenide glasses, and functional polymers [10,11,12,13,14,15,16,17,18]. The abundance of materials and structures have given optical fiber a new path of development to meet the needs of radiation sensors. More and more sensing materials are being drawn into fiber shapes directly, further increasing the accuracy and length range of sensing. In addition, optical fiber sensors are moisture-proof, corrosion-resistant, and anti-electromagnetic interference at most ambient conditions due to the core-cladding-coating structure. Most optical fibers have the core-cladding structure and, further, can be coated with a protective polymer layer to avoid its functional core contacting with water, making the fiber waterproof. Glasses and coating polymer materials demonstrate superior chemical stability even in highly corrosive environment, enhancing optical fiber sensors’ environmental adaptions. Meanwhile, optical fiber sensors are not easily interfered by external electromagnetic fields, because commonly used fiber materials are non-conductive, which can further improve the confidence of sensing.

In this review, the fundamentals of optical fiber-based radiation sensing are briefly introduced. Then, the fabrication methods of all-fiber radiation sensors are summarized and discussed in detail, including the advanced fiber thermal drawing method, the in-fiber microstructure generation method, and micron pulling down (μ-PD). Afterward, the applications of fiber enabled radiation sensors are presented. In the final section, we give a brief conclusion and outlook.

## 2. Fundamentals of Optical Fiber-Based Radiation Sensing

With the increasing demands of radiation sensing in nuclear energy, aerospace, military, and medical fields, optical fiber-based radiation sensors have attracted researchers’ attention. Fiber-based radiation sensors can be divided into two categories according to their working principles, which are intrinsic sensors and extrinsic sensors [4,7]. In the sensing process of an intrinsic fiber radiation sensor, the fiber itself serves as the sensitive element that directly reacts with radiations. The fiber sensor generates signals representing the changes on the fiber, such as structural damage and Bragg wavelength drift, as the result of radiation on materials [19,20,21,22]. For the extrinsic fiber radiation sensors, the fiber does not respond to the radiations directly but serves as a transmission channel connecting the sensor unit and signal processing end [23,24,25,26,27,28]. At present, commonly used intrinsic fiber radiation sensors include damage, fiber Bragg grating (FBG), fiber long period grating (LPG), and scintillating sensors, while the extrinsic sensors work mostly based on scintillating.

**Figure 2:**
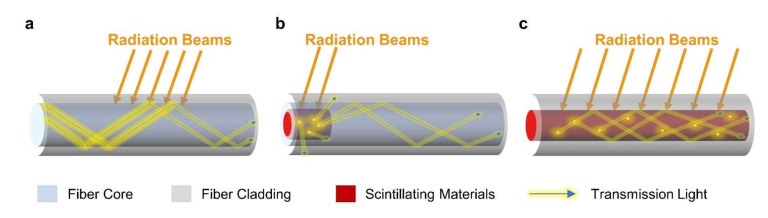
Sensing principles of extrinsic and intrinsic optical fiber radiation sensors. (**a**) Radiation-induced attenuation based optical fiber radiation sensor. (**b**) Radiation-induced luminescence based optical fiber radiation sensor with extrinsic scintillator. (**c**) Radiation-induced luminescence based optical fiber radiation sensor with a scintillating fiber core.

In this section, we will discuss the common sensing principles of extrinsic and intrinsic optical fiber radiation sensors, including radiation-induced attenuation (RIA) (Figure 2a), radiation-induced luminescence (RIL) (Figure 2b,c), and radiation-induced grating wavelength shifting (RI-GWS).

It is worth mentioning that some recent research on artificial intelligence (AI) methods and machine learning methods are used to improve situational awareness, accuracy of data analysis, and control of fiber radiation systems [29,30]. Meanwhile, the neural network type AI can be trained to estimate the radiation in the temporal response, permitting the fiber radiation sensors to be applicable for dosimetry in real time [31].

### 2.1. Radiation-Induced Attenuation (RIA)

#### 2.1.1. Principle of RIA

Radiation at different wavelengths would generate different effects on the fiber materials. Here we take the silica optical fiber as an example. As shown in Figure 2a, when being radiated by the γ rays, the electrons of silica will be ionized to leave with holes. The intrinsic defects, doping defects, impurity defects, and radiation-induced defects can capture these electron-hole pairs to form a specially charged point defect, which is also called the color centers [32,33]. These point defects are able to absorb photons, leading to a change in refractive index and a decrease of the transmission light. Likewise, the α, β, and X-rays interact with silica optical fiber similarly, but their effects vary because of the differences in the energy of rays and the mass of particles. When the radioactive rays pass through the materials, electrons uptake the radiation energy and become free electrons, leaving a hole, and even breaking the original chemical bond when the energy is large enough. The hole can be paired with another electron to form new atomic bonds, resulting in the rearrangement of the atomic structure. If the radioactive rays consist of heavy or high energy particles, the particles could collide with the atomic nucleus and break the atom bonds to form an atomic gap [34,35]. The existence of broken holes, reconstructed atomic structures, and atomic gaps increase the loss of fiber materials while affecting the refractive indexes. Low-power rays cannot provide enough energy to ionize the atoms, introducing defects through electron displacement. For the high-power rays, the electrons are easily ionized, and the atoms also move due to the collision to form defects in the optical fiber. Using the Beer–Lamber law, the radiation-induced absorption growth can be calculated by

(1) $$\mathrm{RIA}\left(\mathrm{dB}/\mathrm{km}\right)=-\frac{10}{L}log\left\{\frac{P_{T}\left(\lambda ,t\right)}{P_{T}^{0}\left(\lambda \right)}\right\},$$

where *L* is the irradiated fiber length, $P_{T}\left(\lambda ,t\right)$ is the measured optical power of the irradiated fiber, *t* is irradiated time, and $P_{T}^{0}\left(\lambda \right)$ is the optical power of the reference fiber [36]. It is worth noting that the point defects or other color centers will not disappear after the radiation is stopped, and that the RIA in optical fibers only decreases with increasing temperature due to the annealing of color centers by heating [37].

The radiation-induced defects and changes of fiber material refractive indexes can be described by the Lorentz–Lorenz formula (Equation (2)) that relates the refractive index of materials to the electronic polarizability of the constituent particles, as well as the Kramers–Kronig relation (Equation (3)), that relates the refractive index to the loss of absorption [38,39]

(2) $$\Delta n=\frac{\left(n^{2}+2\right)\left(n^{2}-1\right)}{6n}\left(\frac{\Delta \rho }{\rho }+\frac{\Delta R}{R}\right),$$

(3) $$\Delta n\left(\lambda \right)=\frac{\lambda^{2}}{2\pi^{2}}\int_{0}^{\infty }\frac{\Delta a\left(\zeta \right)}{\lambda^{2}-\zeta^{2}}d\zeta ,$$

where Δ*n* is the radiation-induced refractive index changes, *ρ* and Δ*ρ* are the density and induced density changes, *R* and Δ*R* is the molar refractivity and its changes, and Δ*a(ζ)* is the photo-bleaching.

Note that, when the total radiation dose becomes large, the degradation of color centers will, eventually, become more significant. It can be observed with the saturation of the RIA value. In addition, RIA value can also be affected by environmental factors, such as temperature. The atoms and molecules move vigorously under a high temperature, which causes the color centers inside the optical fiber to degrade and the RIA value to decrease [40].

#### 2.1.2. Typical Fiber Radiation Dosimeter Based on RIA

**Figure 3:**
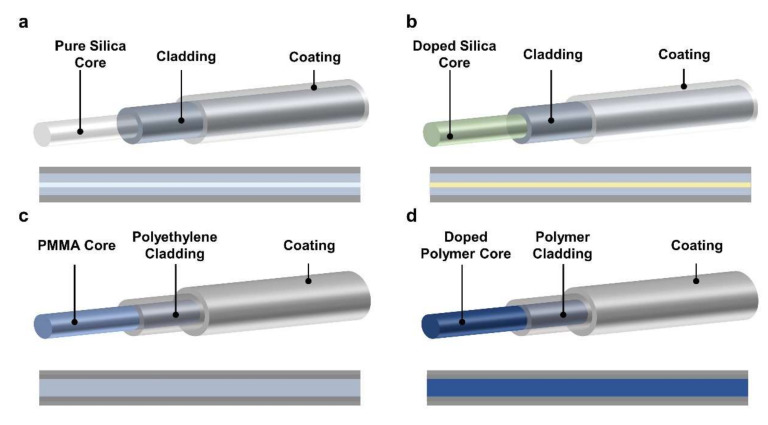
Schematic of widely used fiber radiation sensor based on the RIA principle. (**a**,**b**) silica and doped silica optical fibers. (**c**,**d**) PMMA and doped functional polymer optical fibers.

As shown in Figure 3, many radiation fiber sensors based on the RIA principle have been achieved. As discussed in Section 2.1.1, the silica glass and PMMA can react with radiation rays. These high energy rays impact the atomic structure and atom bonds, leading to the decrease in light transmission. Based on this principle, the commonly used optical fibers based on silica glass are used as radiation sensors (Figure 3a). More recently, some studies have shown that the doped silica has higher sensitivities and radiation resistances, making them promising candidates as radiation sensors, including germanium-doped silica, nitrogen-doped silica, aluminum-doped silica, and phosphorous-doped silica (Figure 3b, Table 1) [21,41,42,43,44,45,46,47,48]. The modifications and changes in the material components can enable optical fibers to be used in different radiation environments. For example, phosphorous-doped silica fiber was verified to be a good candidate for detecting short time X-ray radiation [41]. As an important branch of optical fibers, polymer optical fibers also demonstrate outstanding performance in radiation sensing applications. The polymer optical fibers (POFs) have distinguished optical transmission wavelengths, good ductility, and outstanding mechanical properties [49,50]. The high energy radiation rays can also induce high optical loss into PMMA optical fibers (Figure 3c) [21,47]. Meanwhile, the perfluorinated (PF) POFs are highly radiation sensitive and online radiation monitoring with sensitivity as high as 135 dBm^−1^/kGy (Figure 3d) [48,51]. Therefore, some commercial fiber radiation sensors based on polymer materials are developed for low-cost and real-time radiation monitoring [48].

### 2.2. Radiation-induced Luminescence (RIL)

#### 2.2.1. Principle of RIL

As shown in Figure 2b,c, another conventional method for detecting the radiation dose is to measure the materials scintillation intensity under radiations, including radioluminescence (RL), thermoluminescence (TL), and optically-stimulated luminescence (OSL).

Radioluminescence (RL) is an effect in which atoms are excited into a high-energy state by absorption of radiation energy and then generate photons through spontaneous emission. Some wavelengths of radioluminescence are located at the visible and infrared bands, which can transmit through optical fiber and be collected by the analysis instruments [25,27,52,53]. The radiation power is then calculated by counting the number of photons or measuring the light intensity. The effects of radiation-induced thermoluminescence (TL) and optically stimulated luminescence (OSL) have similar principles [54,55,56]. These effects relate to the impurities that create localized energy levels within the forbidden energy gap. When the TL and OSL materials are ionized by radiation rays, free electrons and holes are excited and then trapped by the lattice defects or impurity in the material during the transition. Heat or light energy can help the excited electrons and holes escape from the traps to combine and stimulate photons. The number of captured electrons before saturation is highly dependent on the radiation dose. The intensities of TL and OSL are also proportional to temperature.

#### 2.2.2. Typical Fiber Radiation Dosimeter Based on RIL

**Figure 4:**
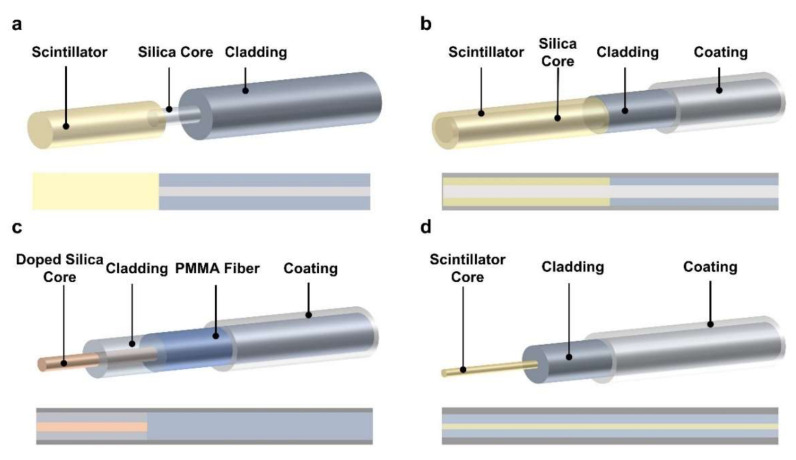
Schematic of widely used fiber radiation sensor based on the RIL principle. (**a**) Scintillator-connected optical fibers radiation sensor. (**b**) Scintillator-covered-optical fibers radiation sensor. (**c**) Limited length RIL radiation fiber sensor. (**d**) All-fiber RIL radiation fiber sensor.

As discussed in the previous section, the RIL includes several detection principles, such as RL, TL, and OSL, based on extrinsic sensing and intrinsic sensing structures. For the extrinsic fiber sensor, a scintillator will be used to react with radiation rays and to emit photons. As shown in Figure 4a–c, the scintillating materials are fixed to the optical fiber end face by bonding, inlaying, or wrapping to detect the radiation rays. Then the generated photons from the scintillating materials are collected by the connected optical fiber and transmitted to the photodetectors. The optical fiber only performs the function of optical signal transmission. The detection of radiation rays is realized by the extrinsic scintillating materials, such as rare earth element doped inorganic scintillators (NaI, CsI, KBr, Gd_2_O_3_, Al_2_O_3_, YVO_4_) and plastic scintillators (Quantum Dots Doped PMMA and Polystyrene), etc. [23,24,25,28,53,57,58,59,60,61]. These extrinsic fiber radiation sensors still have some inherent limitations. On one hand, the optical signal stimulated by radiation rays suffers from optical scattering and other energy loss due to the connecting interface, reducing the accuracy and measurement limits of sensing. On the other hand, the range of measurement is also limited by the length of the scintillating materials, decreasing radiation capture and detection efficiency. Meanwhile, the robustness of this feature needs to be improved.

To improve the radiation-capturing capability, detection length/accuracy, and robustness of devices, all-fiber RIL radiation fiber sensors were developed as intrinsic sensing (Figure 4d). Based on the development of the fiber thermal drawing process and material engineering, scintillating materials, such as rare earth element doped silica, Ce doped YAlO_3_, Ce doped Lu_1.8_Y_2_SiO_3_, etc., are inserted into the fiber cladding tube and drawn into scintillating fibers directly (Table 1) [62,63,64,65,66,67,68,69,70,71,72]. For the intrinsic RIL fiber sensor, the fiber not only transmits the generated photons, but also serves as the light emitting element that directly reacts with radiations.

### 2.3. Radiation-induced Grating Wavelength Shifting (RI-GWS)

**Figure 5:**
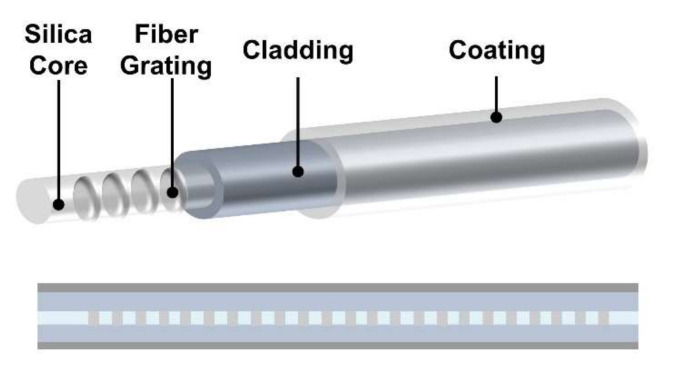
Schematic of fiber grating for radiation-induced wavelength shifting sensing.

As mentioned above, the radiation energy will induce the defect points and crystal structure changes, leading to changes of the refractive index of fiber materials. In addition, the radiation-induced effects also induce the temperature changes based on the thermo-optic parameters, leading to the changes in material density and grating periods [73]. These changes will not only induce the RIA phenomenon but can also lead to a wavelength shifting phenomenon based on fiber Bragg grating (FBG) and long period optical fiber grating (LPG), accordingly (Figure 5) [74,75,76]. The radiation-induced Bragg wavelength shifting (BWS) Δ*λ_B_* can be calculated by

(4) $$\frac{\Delta \lambda_{B}}{\lambda_{B}}=\frac{\Delta n_{rad}}{n_{eff}}+\frac{\Delta \Lambda }{\Lambda },$$

where *λ_B_* is ~1550 nm for FBG, Δ*n_rad_* is radiation-induced effective refractive index, *n_eff_* is the effective refractive index, Λ and ΔΛ are the grating period and grating period changes. As the wavelength shifts of FBG are sensitive to the change of radiation but not to the intensity of radiation, the FBG radiation sensors are suitable for strong radiation scenarios at high temperatures. In 2006, Krebber et al. demonstrated the capability of using FBG fibers for radiation sensing under an intense radiation (>2 kGy) [75]. In 2018, Mas et al. used an FBG fiber to monitor the radiation dose inside the MIT nuclear reactor (MITR) under a temperature higher than 600 °C and the radiation power higher than 1 MeV, which clearly illustrates the potential of using FBG sensors in nuclear power plants monitoring. The Bragg wavelength recorded during the experiment presents a stable shift to the short wavelength at the rate of 0.1 nm/day [77]. Recently, LPG optical fiber sensors have shown a large potential in the field of radiation sensing. In 2015, Sporea et al. proved the sensitivity of 1.2 nm/kGy to gamma exposure based on LPG, manufactured by the melting-drawing method based on CO_2_ laser and assisted by a micro-flame [78]. In 2017, Stancălie et al. reported the real-time monitoring of mixed neutron and gamma flux based on the spectral of LPG, written by the electric arc discharge (EDA) technique [73]. In 2018, a temperature-compensated radiation sensor based on two LPG fiber sensors was developed by Stancălie et al. [79]. In 2020, Berruti et al. achieved an uncoated LPG radiation sensor based on a commercial B-Ge codoped optical fiber for multi-parametric sensing in high radiation environments [80]. The sensitivity of the LPG device to an external factor can be expressed as

(5) $$\frac{\delta \lambda_{res}{}^{\left(n\right)}}{\delta \chi }=\gamma^{\left(n\right)}\times \lambda_{res}{}^{\left(n\right)}\times \left[\frac{1}{\Delta n_{eff}{}^{\left(n\right)}}\times \frac{\delta \Delta n_{eff}{}^{\left(n\right)}}{\delta X}+\alpha_{X}\right],$$

where *χ* is the external parameter, *λ_res_*^(*n*)^ is the resonant wavelength of LPG, *γ*^(*n*)^ is the dispersion parameter of mode *n*, *α_χ_* is the external factor-induced change of the grating period. From the results of the current study, it appears that the fiber radiation sensors based on LPG have a higher sensitivity of radiation dose than the sensors based on FBG [78,79,81].

## 3. Fabrication Method of the Intrinsic Fiber Radiation Sensor

As discussed in Section 2, the optical fiber radiation sensors can be broadly classified into extrinsic and intrinsic sensors. For the extrinsic fiber radiation sensors, the radiation luminescence and radiation damages only occur in the extrinsic sensing part. The shape, length, and connection process of the extrinsic sensing materials limit their application, making them unsuitable for large-scale sensing. For the intrinsic fiber radiation sensors, the radiation luminescence and radiation damages occur in the fiber materials directly, greatly improving the sensitivity and sensing coverage. In this section, we will introduce the advances of the fabrication methods of the intrinsic fiber radiation sensors.

### 3.1. Micro Pulling-Down (μ-PD) Method and Laser Heated Pedestal Growth (LHPG) Method

**Figure 6:**
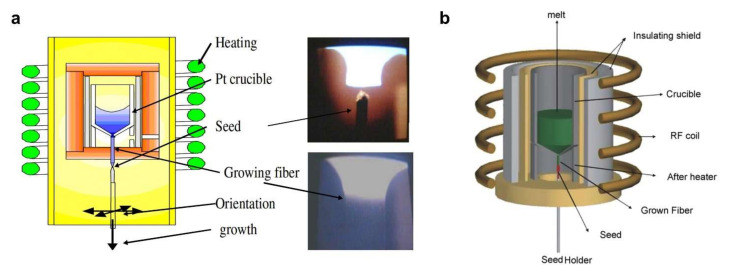
Schematic and photographs of micro pulling down set-up. (**a**,**b**) BGO and YAlO3 crystal fibers grown by micro pulling down technique. Reprinted with permission from ref. [91,99]. Copyright 2009 and 2007, Elsevier B.V.

The single crystals of Bi_2_Ge_3_O_12_ (BGO, melting temperature of 1050 °C), Lu_3_Al_5_G_12_ (LuAG, melting temperature of 1980 °C), Y_3_Al_5_O_12_ (YAG, melting temperature of ~1940 °C), YAlO_3_ (YAP, melting temperature of ~1934 °C) are promising scintillating materials [4]. As shown in Figure 6, the micro pulling-down (μ-PD) method and laser heated pedestal growth (LHPG) method are widely used as crystal growth techniques [91,98,99]. For both methods, a crystal seed is used to guarantee the orientation and crystal structure of the grown crystal. A heating chamber for the μ-PD method or laser heating source for the LHPG method is involved to melt the target materials. Then the crystal seed is pulled slowly and continuously. The new single crystal grows on the liquid/solid interface. Furthermore, to enhance the RIA and RIL effects, rare earth elements, such as Bi, Eu, and Ce, are also doped into these single crystals.

However, the control process of μ-PD and LHPG methods is complex. Many control parameters of systems, such as the heating temperature, the pulling speed, the cooling rate at the liquid/solid surface, and the stability of the system, affect the final quality and length of the grown single crystal. In addition, only specific materials can be prepared by these two methods. Therefore, the restricted material selection, slow crystallization rate, and stringent conditions make the μ-PD and LHPG methods low in productivity, limiting their applications.

### 3.2. Fiber Thermal Drawing Method

**Figure 7:**
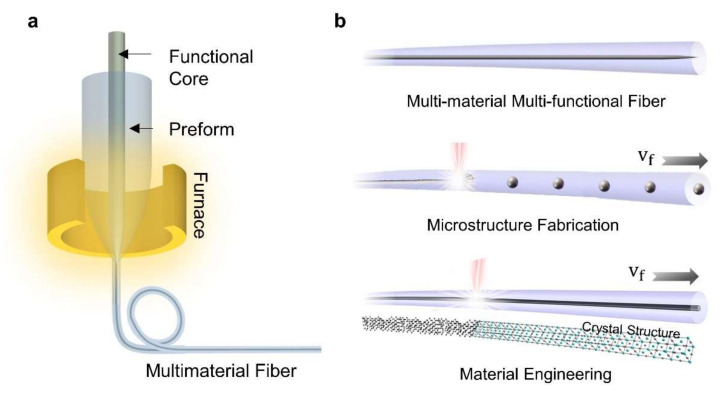
Schematic of multi-material fiber thermal drawing method and in-fiber thermal treatment processing. (**a**) Schematic of advanced fiber thermal drawing process. (**b**) In-fiber microstructure generation process and material engineering process.

Traditional optical fibers are fabricated by the thermal drawing process (Figure 7). First, the doped silica core and cladding are combined to form a macro preform with a diameter of tens of millimeters. Then, the preform is placed on the fiber drawing tower and fed into the heating furnace under a feed-in velocity. The glass preform is softened at high temperature and pulled into a fiber with micron diameter under external tension force. By adjusting the heating temperature, applied force, and pulling velocity, the diameter and mechanical properties of the as-drawn fibers can be precisely controlled.

The commonly used communication optical fibers are fabricated from silica glass and lightly doped silica glass. Recently, with the development of the fiber thermal drawing process and material engineering, the fiber fabrication process provides access to a wider range of materials. For example, this advanced fiber drawing method has embedded metals, semiconductors, chalcogenide glasses, and doped polymers into one multi-material multi-functional fiber, expanding applications of optical fibers to optoelectronic devices, flexible electronics, multiparameter sensing, as well as radiation monitoring.

Several promising attempts have been achieved to fabricate fiber radiation dosimeters based on intrinsic RIL and RIA effects. Without the need for extrinsic sensing units, the fiber thermal drawing process can draw the radiation-sensitive and scintillation materials into an optical fiber directly, with radiation sensing and monitoring capabilities. As shown in Figure 7a, the radiation-sensitive and scintillation materials, such as Ce-doped silica, and Li-enriched glass, are fabricated into the functional fiber core and inserted into the cladding tube to form the multi-material preform. The preform is then drawn into the scintillating fiber with a micron diameter and hundreds of meters in length. The fiber drawing process is accompanied by a large cooling rate and fast pulling velocity, resulting in amorphous or poly-crystalline structures for fiber core.

**Figure 8:**
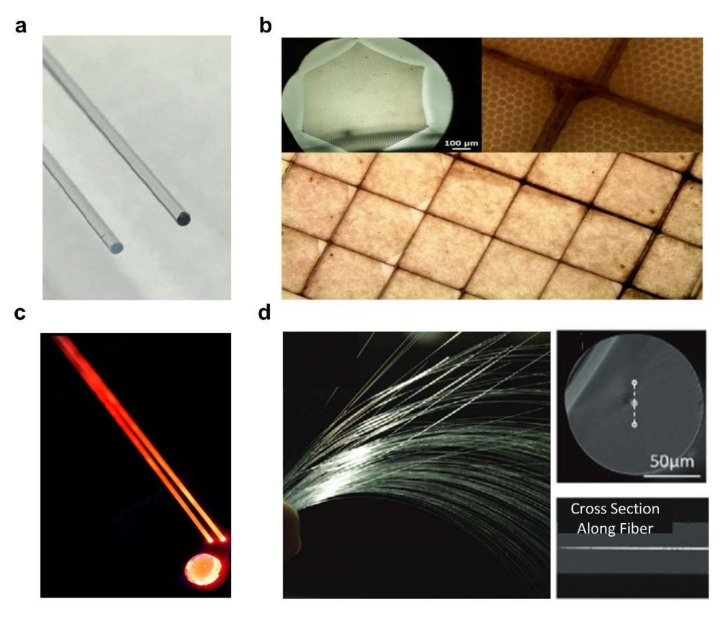
All-fiber radiation sensors. (**a**) 0.05% Ce-doped optical fibers before (on the left) and after 1 kGy irradiation (on the right). Reprinted with permissions from ref. [101]. Copyright 2018, Optical Society of America. (**b**) The cross-section of a Li-glass multicore fiber, and the polished faceplate surface of a multicore array. Reprinted with permissions from ref. [100]. Copyright 2019, IEEE. (**c**) Pr-doped silica scintillating fibers and faceplate. Reprinted with permissions from ref. [70]. Copyright 2017, Elsevier B.V. (**d**) LYSO:Ce core silica cladding scintillating fibers. Reprinted with permissions from ref. [102]. Copyright 2020, WILEY-VCH Verlag GmbH & Co. KGaA, Weinheim, Germany.

In 2018, Cove et al. achieved a cerium-doped sol-gel silica core fluorinated silica cladding fibers for high energy physics applications (Figure 8a). The Ce-doped preform, with a diameter of 10 mm and length of 70 mm, are drawn into continuous and stable fibers at the heating temperature of 1900 K [68]. In 2019, Moore et al. fabricated Li-enriched glass derived multicore scintillating fiber by the fiber thermal drawing process for neutron imaging applications. To increase the neutron detection efficiency, thousands of 6Li enriched silicate glass rods were hexagonally packed within the NKF9 glass tube cladding to form the multicore preform (Figure 8b). The single core diameter of as-drawn multicore fiber was ~8.5 μm and the proof-of-concept faceplate achieved a resolution of ~16 μm for neutron imaging [100]. In 2020, Lv et al. achieved a cerium-doped lutetium-yttrium-oxyorthosilicate (LYSO:Ce) core and silica cladding scintillating fiber by thermal drawing process (Figure 8d). The fiber preform has a diameter of 30 mm and was drawn at a temperature of 2100 °C. The linear attenuation coefficient of the resulting scintillating fiber was 118 times higher than the conventional silica fiber as 68 keV. Based on similar principles and processes, Ce-doped silica, Pr-doped silica, Li-enriched glass and Tb-doped germanate glass and bismuth germanate glass fibers have been generated to achieve all-fiber radiation sensors with high sensitivity (Figure 8).

### 3.3. Methods of In-fiber Microstructure Generation and Crystal Structure Modifications 

The as-drawn fibers fabricated from the fiber thermal drawing methods have a generally polycrystalline or amorphous crystal structure due to the fast drawing speed, which will hinder light transmission, carrier migration, and electrical conductivity of the optical fiber. With the development of the in-fiber processing technology, the thermal treatment processes are used in fiber to fabricate microstructure and modify the crystal structure (Figure 7b).

**Figure 9:**
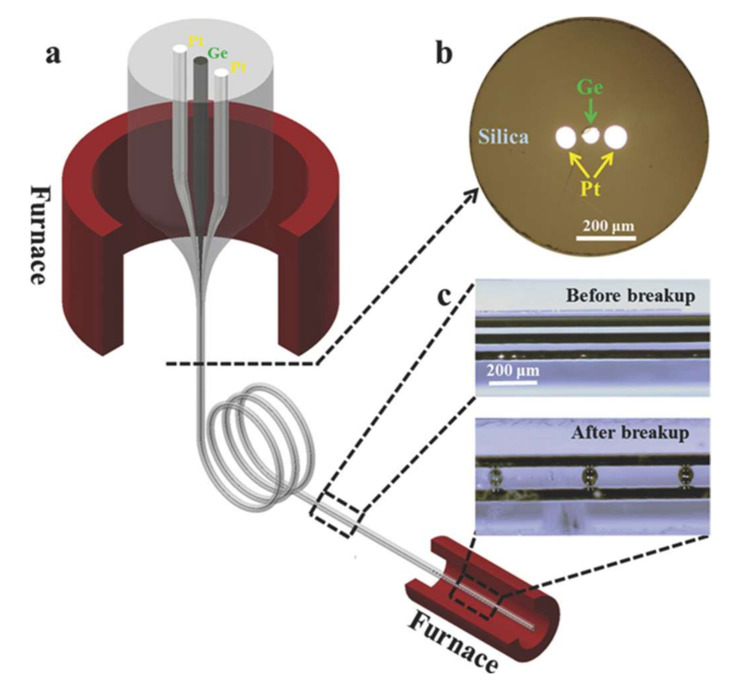
All-in-fiber germanium-platinum optoelectronic sensing units. (**a**) Multimaterial fiber drawn by fiber thermal drawing process. (**b**) Cross-section of germanium-platinum optoelectronic fiber. (**c**) Continuous germanium and platinum fiber cores (top, before breakup) and assembled germanium-platinum micron functional units by thermal induced in-fiber capillary instabilities (bottom, after breakup). Reprinted with permissions from ref. [106]. Copyright 2017, WILEY-VCH Verlag GmbH & Co. KGaA, Weinheim, Germany.

First, a heating source, such as micron tube, oxyhydrogen flame, and laser, is used to soften the fiber. As the cladding materials of fibers are glasses and polymers, they can maintain certain shapes while being softened, providing structural support for the melted fiber cores. The capillary instability will introduce a perturbation at the core/cladding liquid interface due to surface tension force and viscous forces. The development of the perturbation can lead the continuous fiber core to break up into micron and nanoparticles. These in-fiber generated microstructures can further cooperate with other structures in fiber to form multi-functional fibers [11,12,103,104,105]. This intriguing phenomenon prompts us with a new way to fabricate and design novel distributed radiation sensors [16]. For example, the optoelectrical fiber sensors with Ge-Pt and Ce-Cu sensing units (Figure 9) [16,102,106].

Second, the in-fiber thermal treatment process has been proven to be effective for single crystal fiber fabrication. Similar to the LHPG and the laser floating zone (LFZ) methods, we can use a laser as a small and precisely controlled heating source to melt a limited zone of the fiber core. Then, the laser spot scans along with the fiber axial. The fiber core will be melted, resolidified, and recrystallized into single crystal at the liquid/solid interface. The crystal structure and orientation remain consistent throughout the whole fibers. This process should be careful to control some factors, such as laser moving velocity, heating temperature, as well as mechanical and thermal properties of cladding materials. Many single crystal optical fibers have been fabricated by these methods, including silicon, germanium, selenium, tellurium, and gallium antimonide optoelectrical fiber, etc. [12,15,107,108,109,110,111,112,113].

Therefore, the in-fiber thermal treatment process offers a new promising way to fabricate high-efficient single crystal radiation optical fibers with functional microstructures.

## 4. Application

Radiation dosimetry based on optical fibers is being used in many fields. Table 2 shows some mature and possible application fields of optical fiber radiation sensors, such as industry radiation monitoring, radiation monitoring in space, as well as medical radiation dosimetry. In this section, we will introduce some inspiring applications in detail. 

### 4.1. Industrial Radiation Monitoring

**Figure 10:**
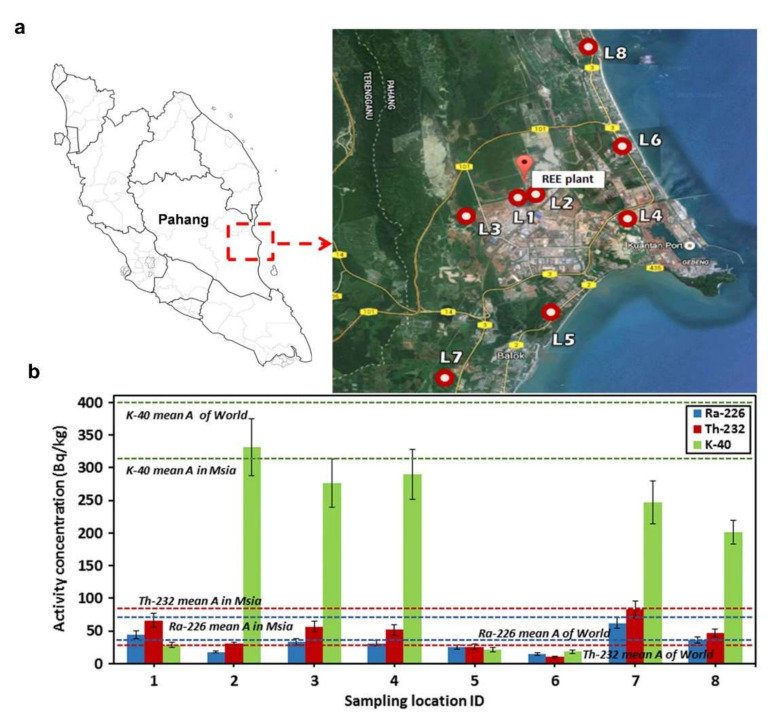
Environment monitoring by silica fiber-based thermoluminescence dosimetry. (**a**) Sampling locations off-site of the rare-earths processing facility, Pahang, Malaysia. (**b**) Activity concentration of elements recorded by the Ge-doped collapsed photonic crystal fiber. Reprinted with permissions from ref. [121]. Copyright 2017, IOP Publishing Ltd.

Optical fiber-based radiation sensors offer adequate sensitivity with their unique ability in distributed radiation measurement; they are small in size, intrinsically insensitive to magnetic fields and electromagnetic interference, and reasonable in cost. These features make them useful in several industrial applications. The fields for optical fiber-based radiation dosimeters to play an important role can be categorized into two types. Optical fiber-based radiation dosimeters are useful in large-scale environmental monitoring for mining plants, mineral processing facilities, and nuclear power plants, where the baseline radioactivity data must be monitored for safety concerns. Therefore, a large area containing a great number of points shall be measured, which is hardly accomplished by commercially available thermoluminescent dosimeters (TLD) due to the cost and manpower limitations, or for measurement in a moving product line. In 2017, Rozaila et al. conducted the assessment for the baseline radioactivity data off-site of a rare earth processing plant in Pahang, Malaysia (Figure 10a) [121]. A Ge-doped collapsed photonic crystal fiber was used as the fiber-form TLD and calibrated against commercially available TLD (TLD-200 and TLD-100). Doses in the range 0.5–10 mGy of several elements were recorded and one point with the radiation higher than the guideline of UNSCEAR safety limit was identified (Figure 10b). For another industrial application, food irradiations for disinfestation, ripening delaying, elimination of pathogenic and spoilage bacteria are widely used today. A high dose of 1 kGy to 10 kGy is delivered in the process, which, thus, raises concerns for the radioactive level on the food, which shall be monitored carefully to enhance consumer confidence. Tanjuddin et al. reported their work to develop optical fibers for food irradiation dosimeters. Ge-B doped optical fibers in different forms and sizes were characterized for their ability in radiation sensing at the kGy level. The linearity, reproducibility, and fading of data recorded by the fiber-based dosimeter give hopes for in-site dosimetry in the food processing line [121].

Another field for optical fiber-based radiation dosimeters to perform is working under extreme conditions, which could be too narrow, too zigzagged, or too dangerous for normal operations. Taking the nuclear power facility as an example, local dose deposition measurements and distributed hot-spots dose monitoring are strongly needed in the nuclear infrastructures, nuclear waste repositories, and the surrounding environment [143]. Optical fiber-based radiation dosimeters are powerful in this case by providing distributed sensors with radiation-tolerant of gamma total doses approaching 1 GGy and neutron doses of up to 1024 n/m^2^ (>0.1 MeV) [144]. Fernandez et al. investigated three optical fiber-based radiation sensors under intense gamma radiation. A fiber-coupled optically stimulated luminescence system, and a scintillation fiber-optic radiation monitoring system was tested for the real-time radiation monitoring at Gy/h level doses rate. A commercial plastic fiber was also examined to measure the radiation with the threshold between 100–1000 Gy; the results showed that PMMA optical fiber can be used as the gamma dosimeter. All fiber-based radiation sensors have met most of the requirements from monitoring a thermonuclear facility [116].

### 4.2. Radiation Monitoring in Space

**Figure 11:**
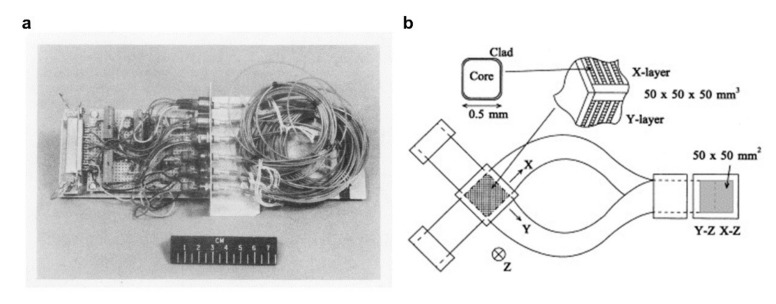
The optical fiber radiation dosimeter for radiation monitoring in space. (**a**) Testbed with sensing fibers for NST-2 mission. Reprinted with permissions from ref. [145]. Copyright 1969, IEEE. (**b**) Structure of a scintillating fiber stack. Reprinted with permissions from ref. [147]. Copyright 2001, Elsevier Science B.V.

Monitoring high-energy cosmic rays is strongly needed in both manned and unmanned spacecraft for health concerns of astronauts and the normal function of the carried instruments. Indeed, dosimeters are also carried as a scientific payload to measure the cosmic rays. Differing from dosimeters used on earth, developing a dosimeter for spacecraft shall minimize the weight and volume as much as possible; they should be able to work normally under the total radiation doses of a few kGy, high vacuum, and extremely low temperature. Optical fiber-based radiation dosimeters have been considered for use in space, because they can be fully embedded in satellite structures to provide a wide field of detection with little impact on the host satellite. The Navigation Technology Satellite 2 (NTS-2) was launched in 1977 carrying a glass fiber as the dosimeter, which firstly illustrated the feasibility based on the darkening of glass (Figure 11a). The system recorded doses of 0.35, 6, and 60 Gy under different aluminum shielding, 367 days after launch [145]. Los Alamos National Laboratory reported two space fiber-optic X-ray burst detectors designed for the STEP mission 3 spacecraft. The first one was made of a 500 μm (diameter) × 4 m (long) scintillating fiber coupled to a silicon photodiode and the second one was a 100 μm core × 1.25 m long multimode Ge-doped silica fiber, which worked based on the radiation darkening phenomenon. The scintillating fiber gave a fast signal for timing and the Ge-doped fiber darkened for a few milliseconds after a burst of X-ray. The coincidence of two signals represented an occurrence of an X-ray burst [146]. Terasawa et al. developed a camera made of a scintillating fiber stack for dosimetry in spacecraft. The camera was able to identify charged particles, neutrons, and gamma-rays in three dimensions (Figure 11b). Especially for neutron dosimetry, the smaller size of fiber helped the reduction of threshold [147]. Most recently, iXblue deployed two several-kilometer-long optical fibers as active dosimeters to measure ionizing radiation in the international space station in 2021, and the first result is waiting for further announcement [148].

### 4.3. Medical Applications

**Figure 12:**
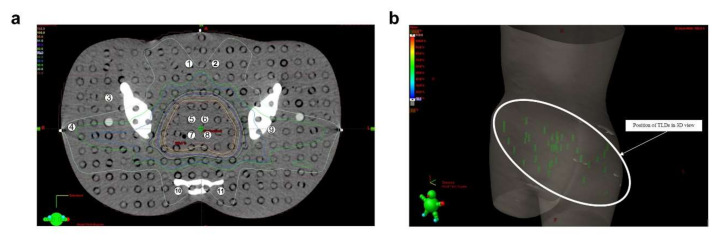
Ge-doped optical fiber for in-vitro. (**a**) Ge-doped optical fibers and LiF TLDs (highlighted) in 3D view. (**b**) Numbers correspond to the positions of Ge-doped optical fibers and LiF TLDs in a Rando-phantom CT slice. Reprinted with permissions from ref. [132]. Copyright 2010, Elsevier B.V.

Optical fiber-based radiation dosimeters play a unique role in radiotherapies majorly due to their ability in high-resolution dose mapping. Before the patient undergoes radiotherapies, accurately evaluating the dose at each point is necessary for designing the treatment plan. Especially for an advanced delivery technique such as intensity modulated radiation therapy (IMRT), in which the dose calculation is more complex. Among numerous materials used for medical radiation dosimetry, thermoluminescent (TL) silica optical fibers have attracted attention for their high spatial resolution, small size, water and corrosion proof, and reasonable cost. Noor et al. demonstrated an in vitro study by using a commercial TL Ge-doped silica fiber for IMRT dose mapping in three dimensions. The fiber verified high and low dose regions in the treatment planning system for 6 MV and 15 MV normal photon energy, and the result showed good agreement with the conventional LiF thermoluminescent dosimeters (Figure 12) [132]. Another study conducted by Issa et al. demonstrated the feasibility of using Ge-doped silica optical fiber for brachytherapy source dosimetry. The fiber offered a sub-mm spatial resolution, a linear response from 10 cGy to >1 kGy, and dose-rate independence. The measurement data showed good agreement to simulations and dose mapping of the treatment planning system [136].

## 5. Conclusion and Outlook

In this review, we discuss the current state of optical fiber-based radiation sensors, including extrinsic sensors that rely on extrinsic materials and structures as sensing units, as well as intrinsic fiber sensors that take fiber as sensing units. The basic sensing principles of fiber-based radiation sensors were introduced. The underlying mechanism and actual phenomena of RIL, RIA, and RI-GWS were presented. Various sensor structures based on different sensing principles were also summarized. Meanwhile, the fabrication processes of the intrinsic fiber radiation sensor were introduced. The single crystal scintillating fiber can be fabricated from the micro pulling down process and laser heating pedestal growth. The advanced multi-material scintillating fibers can be drawn directly based on the fiber drawing process. Further in-fiber microstructure generation and crystal structure modifications were also introduced. In addition, various applications were reviewed, including radiation monitoring in industry and space, industrial radiation process, and medical treatment and monitoring. The fiber-based radiation sensors can measure radiation doses from μGy to kGy, covering a large spatial scale with high resolution. They also can be bundled to form a sensing faceplate for radiographic imaging with micron pixels.

Despite the promising advantages in applications, optical fiber-based radiation sensors still have room for improvement. The optical fiber sensing for radiation is sometimes nonlinear, which will affect the calibration of specific radiation values and the monitoring accuracy. In addition, the measurement results are not decided by the radiation intensity alone. Other environmental factors, such as temperature, humidity, and external pressure, can all have an impact on the final detection result. The decoupling of the different influencing factors needs to be further investigated. Meanwhile, the spatial and temporal resolution of optical fiber based radiation sensors also needs to be further improved and optimized.

For future development, we offer some possible options. First, the uniformity of fabrication process and testing standard of fiber-based radiation sensors needs to be established. Past studies have shown that the sensing performance shows variability even for two fibers of the same type from the same company [81]. The fiber fabrication tolerance is the main reason for this difference. Meanwhile, the fabrication of FBGs and LPGs also introduces some slight differences in grating periods, which will further disrupt the uniformity of the fiber-based radiation sensor performance. On the other hand, there is no uniform method or standard for radiation testing. Many environmental factors can be coupled with the radiation testing results, further affecting the accuracy. Nowadays, there have been many attempts to improve the accuracy and repeatability of calibration testing. Second, distributed optical fiber sensors (DOFS) are a tremendously advantageous application of fiber optic sensing [149,150]. DOFS can provide dense and accurate sampling points over long distances at a low cost. The distributed acoustic sensor (DAS), and distributed temperature sensor (DTS) have been used widely in practice for seismic monitoring, geothermal sensing, building quality monitoring, and other fields. Several existing fiber-based radiation sensing technologies have the potential for distributed sensing. The broad application prospects of distributed radiation sensors (DRS) make it a worthy direction for development to improve the spatial and temporal resolution of the optical fiber radiation sensors. Last, the multi-material multi-functional fibers are a new and promising way for optical fiber-based radiation sensors. From one side, the abundance of fiber optic coating and encapsulation materials can provide additional sensing mechanisms. Rather than optical effects, the sensing mechanisms based on the fiber coating, such as optoelectric effect and track elastic effect, can offer more solutions [151]. On another side, with the advanced fiber thermal drawing process, plenty of materials, not just traditional optical fiber materials, could be drawn into the fiber shapes, providing more suitable materials for radiation sensing. Meanwhile, the multi-material optical fibers can be fabricated with different materials together into one fiber, including but not limited to optical materials, thermoelectric materials, and piezoelectric materials. This composite of materials and functions shows a direction for the signal decoupling.

## Figures and Tables

**Table 1:** Fiber-based Radiation Sensors.

Principle	Structure	Material	Ionizing Radiation	Refs.
**Radiation-induced Attenuation (RIA)**	Intrinsic Fiber Sensor	P Doped and P/Ce Codoped Silica	X-ray, Gamma-ray, and Beta Particle	[42,43,44,45]
		Al Doped Silica	X-ray (0–500 Gy), and Beta Particle	[44,46]
		N Doped Silica	X-ray	[41]
		PMMA	Gamma-ray	[21,47]
		Perfluorinated Polymer	Gamma-ray (0–100 Gy)	[48]
**Optically Stimulated Luminescence (OSL)**	Extrinsic Sensor Connected to Fiber	Eu Doped KBr	Gamma-ray	[57]
	Intrinsic Fiber Sensor	Ge Doped Silica	Beta Particle	[82]
		Fluoride Phosphate Glass	Beta Particle	[83,84]
**Thermo-Luminescence (TL)**	Intrinsic Fiber Sensor	Nd Doped Silica	X-ray	[62]
		Ge Doped Silica	Gamma-ray, Alpha Particle, and Proton Beam	[63,64,65,66]
		Al Doped Silica	Alpha Particle	[65]
		Al-Tm Codoped Silica	Gamma-ray	[67]
**Radio-Luminescence (RL)**	Extrinsic Sensor Connected to Fiber	Tb Doped Gd_2_O_2_S	X-ray and Beta Particles	[28,53,58,59,60]
		Tb/Ce Codoped Gd_2_O_3_	X-ray	[61]
		Cr Doped Al_2_O_3_	Gamma-ray and Beta Particle	[23,24,25]
		Eu Doped YVO_4_	Gamma-ray	[24]
		Ti Doped CsI	Gamma-ray and Beta Particle	[24,28]
		Eu Doped and Eu/Li Codoped Y_2_O_3_	Gamma-ray	[24,26,27]
		ZnWO_4_	Beta Particle	[28]
		Quantum Dots Doped PMMA	X-ray (20–500 cGy)	[85]
		Polystyrene	Gamma-ray	[86]
	Intrinsic Fiber Sensor	Ce Doped Silica	X-ray and Proton Beam	[68,69,87]
		Pr Doped Silica	X-ray and Gamma-ray	[70]
		Tb Doped Silica	X-ray	[88]
		Gd Doped Silica	Proton Beam	[89]
	Extrinsic Sensor Connected to Fiber and Intrinsic Fiber Sensor	Bi_2_O_3_-GeO_2_ (BGO)	X-ray	[90]
		Ce Doped YAlO_3_ (YAP)	Gamma-ray	[91]
		Ce Doped Y_3_Al_5_O_12_	Beta Particle	[28]
		Lu_3_Al_5_G_23_	Gamma-ray and Proton Beam (0–100 kGy)	[71,72]
		Ce Doped Lu_1.8_Y_2_SiO_5_ (LYSO)	Gamma-ray	[92]
**Radiation-induced Grating Wavelength Shift (RI-GWS)**	Fiber Bragg Grating	Ge/B Codoped Silica	Gamma-ray (0–116 kGy)	[93]
	Long Period Grating	Ge/B doped or Pure Silica Core and F Doped Cladding	Gramma-ray	[78,79,80,81,94,95,96,97]

**Table 2:** Applications of Optical Fiber Radiation Sensors.

Application		Ionizing Radiation	Refs
**Industry Radiation Monitoring**	Food Irradiation Dosimetry	X-ray, Gamma-ray	[114,115]
	Monitoring of Nuclear Industry Nuclear Waste Storage Nuclear Reactor Core	Gamma-ray	[116,117,118,119,120]
	Environmental Monitoring	X-ray, Gamma-ray	[121]
**Radiation Monitoring in Space**	Dosimetry for Spacecraft Shall	X-ray, Gamma-ray	[20,122,123]
	Radiation Monitoring	X-ray, Gamma-ray	[124,125,126]
**Medical Radiation** **Dosimetry**	External Beam Radiotherapy	X-ray, Gamma-ray, Proton beams	[127,128,129,130,131,132,133]
	Brachytherapy	X-ray, Gamma-ray, Beta radiation	[134,135,136,137,138]
	Diagnostic Radiology	X-ray, Gamma-ray	[139,140,141,142]

## Data Availability

Not applicable.
